# Kounis Syndrome Secondary to Laxative Administration

**DOI:** 10.1155/2022/6087176

**Published:** 2022-06-22

**Authors:** Mateo Zuluaga-Gómez, Daniel González-Arroyave, Carlos M. Ardila

**Affiliations:** ^1^Hospital San Vicente Fundación, Rionegro, Colombia; ^2^Universidad Bolivariana, Medellín, Colombia; ^3^Universidad de Antioquia, Medellín, Colombia

## Abstract

Kounis syndrome (KS) is defined as an acute coronary syndrome triggered by the release of inflammatory mediators after an allergic attack. It usually occurs secondary to allergic injuries from foods, medications, and insect bites. However, there are no known reports of KS secondary to the intake of laxatives. This article reports the case of a 43-year-old woman who, after ingesting a dose of sodium phosphate monobasic/sodium phosphate dibasic, presented a maculopapular rash on the trunk and extremities. The electrocardiogram showed ST depression in V4-V5-V6 and signs of prolonged QTc; troponin I uptake was positive. Due to presumed myocardial injury and high suspicion of coronary disease, coronary angiography was requested, which showed epicardial coronary arteries, without angiographically significant stenosis, thus confirming the presence of KS secondary to the ingestion of a laxative.

## 1. Introduction

Kounis syndrome (KS) is defined as an acute coronary syndrome that presents as unstable angina that may be secondary to vasospastic or nonvasospastic etiology, and even as a manifestation of acute myocardial infarction, triggered by the release of inflammatory mediators after allergic aggression [1]. It has been reported that there are three types of KS associated with the same number of different pathophysiological processes derived from the activation and degranulation of mast cells, which lead to the release of powerful inflammatory mediators. Thus, type I is a coronary spasm in patients with normal or near-normal coronary arteries [1,2]; established or pre-existing coronary artery disease in which inflammation leads to coronary vasospasm or plaque rupture (type II); or in-stent thrombosis and restenosis (type III) [2]. This syndrome generally occurs secondary to allergic injuries from foods, medications, and insect bites [2, 3]. Anaphylactic reactions to polyethylene glycol are very rare [4], and there are no known reports of KS secondary to this type of medication.

## 2. Case Report

A 43-year-old female patient was referred to a high-complexity hospital for the suspected coronary syndrome. The patient reported a history of chronic low back pain and cholelithiasis, waiting for scheduling and management by general surgery on an outpatient basis.

In the initial care hospital and due to her low back pain, a simple X-ray of the total spine was sent and a laxative preparation was ordered for this procedure. One dose of sodium phosphate monobasic/sodium phosphate dibasic (Travad oral®) was administered. Fifteen minutes after ingestion, the patient presented a maculopapular rash on the trunk and extremities, an event that later became generalized; this situation was associated with dyspnea and use of accessory muscles, in addition to diarrheal stools on multiple occasions. Subsequently, the patient consults again and was assessed in the emergency room, where the following vital signs were found: blood pressure 88/64 mm Hg, heart rate 122 bpm, respiratory rate 32, and oxygen saturation 88–89%. It was then interpreted as anaphylaxis, for which management was started with IM adrenaline until a total dose of 3 mg was reached. With this, improvement in blood pressure figures and control of skin and gastrointestinal symptoms were obtained.

**Figure 1:**
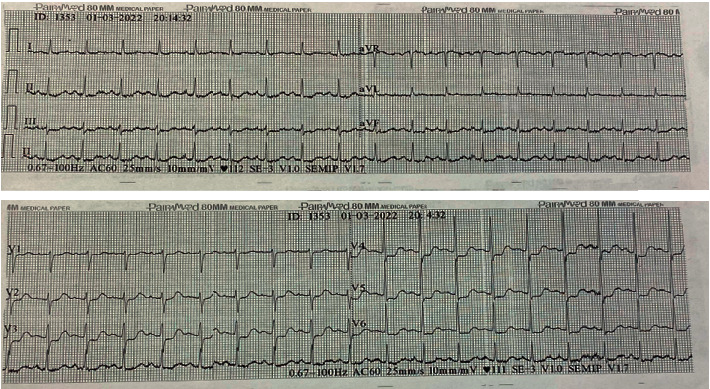
Initial electrocardiogram of the referral site. Sinus rhythm and ST-segment depression in V4-V5-V6 (lateral aspect).

**Figure 2:**
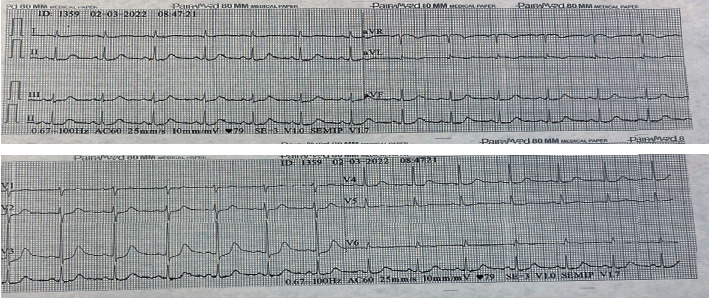
Initial electrocardiogram of the referral site (posterior leads). Sinus rhythm and extension of ischemia to the posterior face of the heart is ruled out.

During her observation in the emergency department, the patient reported a sensation of chest discomfort, epigastric pain, and abdominal pain (after the administration of adrenaline); therefore, this was considered as a diagnostic challenge/differential. It was decided to perform an electrocardiogram in which ST depression was interpreted in V4-V5-V6 (Figures 1 and 2). Troponin I (ultrasensitive) was also taken, which was positive for the cutoff point (4.8 ng/ml; cutoff up to 0.3 ng/ml). Management was then started with aspirin 300 mg, atorvastatin 80 mg, furosemide 40 mg every 8 hours intravenously (IV), isosorbide dinitrate in case of angina, methylprednisolone 50 mg every 8 hours IV, and enoxaparin 60 mg subcutaneously every 24 hours.

**Figure 3:**
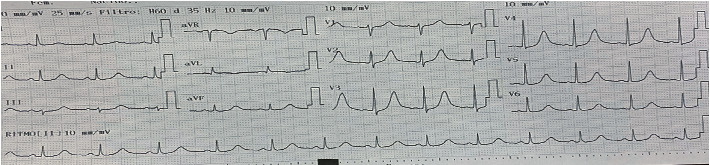
Initial electrocardiogram in a high-complexity hospital. Sinus rhythm, heart rate 75 bpm, QTc 521, QRS 70, PR 154, and prominent precordial T waves.

Subsequently, a referral was made to a hospital with a higher level of complexity due to a diagnosis of non-ST elevation myocardial infarction, with Killip III classification, due to crepitus and radiological signs of acute pulmonary edema. Upon her admission, there was no clear clinical evidence of angina or chest pain, she denied paroxysmal dyspnea, denied bendopnea, denied extremities edema, denied other symptoms, and reported improvement of the rash. She presented the following vital signs at that time: blood pressure 122/74 mm Hg, heart rate 78 beats per minute (bpm), respiratory rate 18 per minute, and oxygen saturation 98%. On physical examination, no use of accessory muscles, no respiratory effort, rhythmic heart sounds, and no presence of heart murmurs were observed. An electrocardiogram was taken, which reported sinus rhythm, heart rate of 79 bpm, QTc 521, QRS 70, PR 154, and prominent precordial *T* waves (Figure 3).

With the above, and due to signs of prolonged QTc, magnesium sulfate was administered, 1 gram every 8 hours IV. Moreover, due to suspicion of a coronary syndrome, high-sensitivity troponin (hs-cTN) was requested again, presenting a value of 10854, that is, positive. Furthermore, leukocytes 16,200/ul, neutrophils 78.9%, hemoglobin 12.1 g/dL, platelets 259,000/ml, INR (international normalized ratio) 1.0, potassium 3.8 mmol/L, magnesium 1.8 mg/dL, sodium 140 mmol/L, nitrogen urea 13.4, creatinine 0.7, alanine aminotransferase 16 IU/L, and aspartate aminotransferase 55 IU/L.

**Figure 4:**
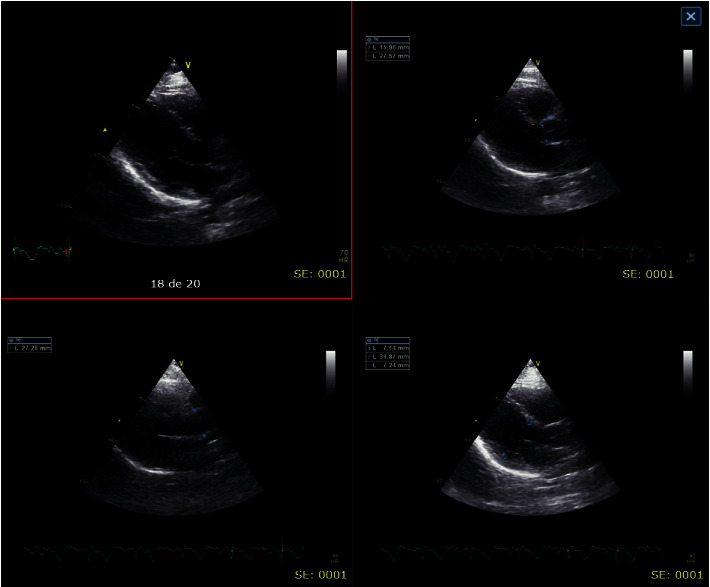
Transthoracic echocardiogram.

**Figure 5:**
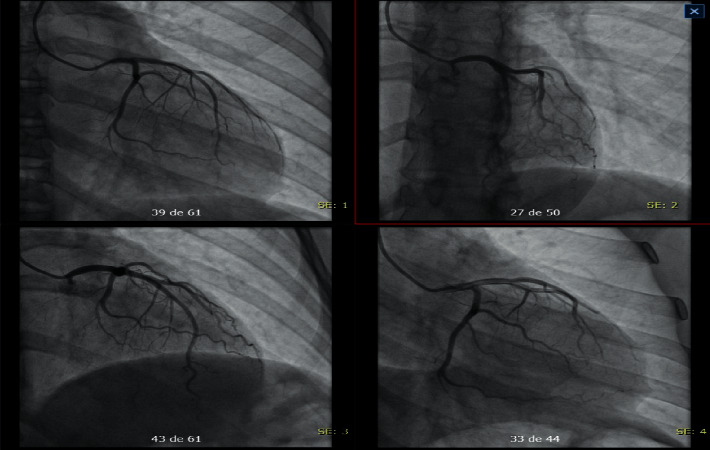
Coronary arteriography.

Due to electrocardiographic findings and positive troponin (biomarker), a transthoracic echocardiogram was requested (Figure 4), reporting a normal-sized left ventricle, moderate hypokinesia of the middle segment of the anterior and lateral wall, normal contractility of the rest of the segments, LVEF (left ventricular ejection fraction) 55%, no diastolic dysfunction, the right ventricle of normal size and systolic function, TAPSE (tricuspid annular plane systolic excursion): 21 mm, nonquantifiable pulmonary systolic pressure, valvular apparatus without relevant alterations, size atria normal, pericardium and great vessels without relevant alterations, IS/IVS without defects or shunts, no intracavitary thrombi, and normal in the rest of the study. Due to presumed myocardial injury and high suspicion of coronary artery disease, coronary angiography was requested, which showed epicardial coronary arteries, without angiographically significant stenosis (Figure 5), thus confirming the presence of Kounis syndrome. The patient was discharged 3 days after admission, asymptomatic, without complications, with outpatient cardiology follow-up, and secondary cardiovascular prevention started (acetylsalicylic acid 100 mg daily, atorvastatin 40 mg nightly).

## 3. Discussion

The cardiovascular system can be highly compromised in the presence of allergic reactions. This is how a dangerous and serious cardiac manifestation such as KS can occur, which can include potential symptoms such as hypotension, arrhythmias, and ventricular dysfunction [5–7]. Inflammatory cells, including eosinophils, macrophages, and mast cells, play a relevant role in hypersensitivity reactions. They start an inflammatory cascade through the release of leukotrienes, thromboxane, IgE, tryptase, histamine, and cytokines, which act on different organic systems [8]. At the coronary level, histamine can cause vasoconstriction, which seems to be the main pathophysiological mechanism in KS [9].

In the present case, a KS secondary to the administration of a laxative is described. Although the literature reports KS secondary to drugs [2,3], especially antibiotics [2], there is no known report related to this type of medication. However, the clinical manifestations related to KS were similar to those presented in the patient reported in this case. Chest pain has been reported to be the most common sign, followed by allergy-related symptoms (pruritus and edema) [2]. Despite this, patients admitted to the emergency department without clear cardiac symptoms have been documented, but that the search for etiology and phenomena of cardiac ischemia are documented either due to changes in the electrocardiogram or due to elevation of biomarkers [10]; the presentation of patients with the acute coronary syndrome as the only manifestation of a hypersensitivity reaction is less frequent [11]. Due to this wide range of symptomatologic variants and the absence of cardiac risk factors, KS remains underdiagnosed [2].

Different electrocardiographic patterns associated with KS are presented in the literature, including ST-segment elevation in the anterior and inferior face of the electrocardiogram, followed by repolarization disorders, heart block, atrial fibrillation, and ventricular ectopy rhythms [12].

Allergy studies include the measurement of serum levels of tryptase, histamine, immunoglobulin E, complement levels, and eosinophil count. However, these studies are not as widely available. Therefore, among all patients with angina and a suspected allergy component, and the coronary angiography is normal, or indicates that there are no coronary arteries with lesions, and the KS picture is more suggestive. Troponin I usually presents elevation in 7–10% of patients with anaphylaxis, without having such a high range, as in patients with coronary syndrome due to atherothrombosis [13, 14].

Regarding the management of KS, a great versatility between cases has been described, possibly due to the absence of predetermined algorithms. However, the recommended therapy consists of concomitant management of myocardial ischemia and allergic reaction [2]. The use of nitrates should be considered as long as the patient does not present some degree of hypotension, improving the signs and symptoms of vasospasm. It is not clear the use of acetylsalicylic acid as well as inhibitors of the glycoprotein IIb-IIIa complex in KS; however, until the active coronary disease has been ruled out, it should be recommended if the initial clinical picture is more in favor of an acute coronary syndrome. The use of other medications such as adrenaline, steroids, and antihistamines is recommended under the pretext of the distributive shock that these patients present, as long as they are indicated [15, 16].

Finally, it is important to keep in mind that hypersensitivity reactions, regardless of the primary trigger, can be complicated by the acute coronary syndrome.

## Data Availability

The clinical data utilized in this report are described in this article.
